# Time-Space Translation: A Developmental Principle

**DOI:** 10.1100/tsw.2010.208

**Published:** 2010-11-04

**Authors:** A. J. Durston, H. J. Jansen, S. A. Wacker

**Affiliations:** ^1^ Sylvius Laboratory, Leiden, The Netherlands; ^2^ Institute of Biochemistry, University of Ulm, Germany

**Keywords:** Hox genes, gastrulation, early development, Xen

## Abstract

We review a recently discovered developmental mechanism. Anterior-posterior positional information for the vertebrate trunk is generated by sequential interactions between a timer in the early nonorganizer mesoderm (NOM) and the Spemann organizer (SO). The timer is characterized by temporally collinear activation of a series of Hox genes in the early ventral and lateral mesoderm (i.e., the NOM) of the Xenopus gastrula. This early Hox gene expression is transient, unless it is stabilized by signals from the SO. The NOM and the SO undergo timed interactions due to morphogenetic movements during gastrulation, which lead to the formation of an anterior-posterior axial pattern and stable Hox gene expression. When separated from each other, neither the NOM nor the SO is able to induce anterior-posterior pattern formation of the trunk. We present a model describing that the NOM acquires transiently stable hox codes and spatial collinearity, and that morphogenetic movements then continually bring new cells from the NOM within the range of SO signals that cause transfer of the mesodermal pattern to a stable pattern in neurectoderm and, thereby, create patterned axial structures. In doing so, the age of the NOM, but not the age of the SO, defines positional values along the anterior-posterior axis. We postulate that the temporal information from the NOM is linked to mesodermal Hox expression. The role of the SO for trunk patterning turns out to be the induction of neural tissue as prerequisite for neural hox patterning. Apparently, development of a stable anterior-posterior pattern requires neural hox patterning. We believe that this mechanism represents a developmental principle.

## INTRODUCTION

While still at school, most of us are deeply impressed by the underlying principles that so beautifully explain why the chemical elements are ordered as they are in the periodic table and may wonder, with the influential innovator Brian Goodwin[1], whether there might be equally powerful principles that account for the awe-inspiring diversity of body forms in the living realm. In fact, the question of how an organism acquires its structure and form during embryogenesis is one of the most intriguing and challenging in Science. It is now becoming clear that there are, indeed, universal principles.

The principle that has received most attention is the concept of a diffusion gradient. An informational molecule (morphogen) is synthesized locally (perhaps by an appropriate dynamical system), diffuses from the source, and is read off for values above different threshold concentrations to turn on different genes, behaviors, or differentiation states. The idea is depicted by Lewis Wolpert's French flag analogy[2,3], where a one-dimensional gradient generates the three color zones of the French flag. The morphogen can be an intracellular protein, e.g., a transcription factor, but is more usually a low-molecular-weight signal molecule. Although this idea has been proposed and evidence has been presented in a large number of cases, there are only a couple of cases where the evidence is unambiguously convincing. Coincidentally, one of these concerns the early specification of the main head-tail body axis in the fruitfly *Drosophila* by a gradient of the transcription factor bicoid[4]. It is interesting that this mechanism does not represent a general principle. Whereas in *Drosophila* this anterior-posterior patterning mechanism acts during a static phase of development, anterior-posterior patterning in vertebrates is established during gastrulation, a highly dynamic period with a series of complex morphogenetic movements. It is shown below that a vertebrate makes its main body axis by a different mechanism. As discussed below, there are developmental principles that rely on the dynamics of the developing system; namely, timing mechanisms. The sections that follow discuss a novel dynamic mechanism that patterns the trunk region of the vertebrate main body axis. This mechanism has three aspects: temporally collinear expression of Hox genes in gastrula nonorganizer mesoderm (NOM); interaction between the NOM and the Spemann organizer (SO); vertical signaling from gastrula NOM to neurectoderm.

## THE HOX GENES

The Hox genes are a transcription factor gene subfamily that is famously implicated in mapping out the main body axis of animals throughout nearly the whole animal kingdom. In vertebrates, Hox genes are closely clustered in genetic complexes that show temporal and spatial collinearity. The time and (axial anterior-posterior) position of expression of each hox gene correlate with its genetic position in the complex. The most 3' gene is expressed first and most anteriorly, and the most 5' gene last and most posteriorly. It has been argued that temporal collinearity is the primary property. This close clustering and collinearity is a rare characteristic in evolution. In its extreme form, it is limited to the vertebrates[5]. Hox collinearity is at the center of modern thinking about vertebrate hox genes. It obviously provides a potential mechanism for patterning hox-expressing structures like the main body axis and the developing limb. Although this aspect is clearly very important, the question of how temporal collinearity could pattern an axis has not received much attention. There are a few ideas about this. Duboule proposed that localized growth control could combine with temporal collinearity to pattern an axis in his "Einbahnstrasse" model[6]. Iimura and Pourquié proposed that this patterning occurs via temporally collinear control of gastrulation movements by Hox genes[7], but there is very little research into these concepts, or any detailed mechanistic explanation or model as to how hox temporal collinearity could pattern an axis. Nearly all extant models concern morphogen gradients and use hox genes only as individual targets of gradient morphogens (e.g., [8,9,10,11]). The purpose of this article is to review and discuss studies that have investigated the connection between Hox collinearity and axial patterning. These studies led to a specific model featuring time-space translation and an interaction between the hox-expressing NOM and the SO.

## HOX GENES IN GASTRULATION

The early vertebrate embryo undergoes a drastic morphogenetic reorganization leading to its definitive body plan during a process called gastrulation. In Amphibia, three tissue types – ectoderm, mesoderm, and endoderm – which originally form parts of the surface of a hollow spherical embryo, become reorganized into layers with endoderm inside, ectoderm outside, and mesoderm in between. This process involves internalization of the mesoderm into the embryo. This internalization occurs more extensively at one side of the embryo (dorsal side) than the other (ventral side), and contributes to subsequent extension of the embryo to an elongated form. This involves a specialized movement called convergence extension.

There are early dorsoventral differences in the embryo, which cause the *Xenopus* gastrula to contain two kinds of mesoderm: the SO, which is dorsal, and the NOM, which is ventrolateral. At one stage, it was thought that the SO provided the anterior-posterior positional information. Spemann published a paper in 1931 that proposed head and tail organizers[12] and there were even proposals of head, tail, and trunk organizers[13,14,15,16]. More recently, it has become clear that, while there is a head organizer that encodes head specificity, by blocking Wnt signaling[17] and a trunk organizer that does not block Wnt, at least some of the spatial information in the posterior (trunk), hox-expressing part of the axis comes from the NOM and its derivatives[9,10,18,19]. We show below, in fact, that the trunk organizer is not informational for anterior-posterior positional values. In general, it has become clear now that an important aspect of the genesis of the anterior-posterior pattern is the transfer of anterior-posterior information from the NOM to the neurectoderm by so-called vertical signaling during regionalization of the central nervous system[9,10,16,18]. We conclude (below) that the trunk organizer provides no positional information, but enables transfer of this information from the NOM to the neurectoderm.

There is a comparable early pattern of hox expression in gastrulae of each of the families of vertebrates that have been well studied, namely fish (zebrafish)[20], amphibians (*Xenopus*)[19], birds (chicken)[21], and mammals (mouse)[22]. The following paragraphs describe the findings in *Xenopus*, the best-studied vertebrate embryo[19,23].

There is a temporally collinear sequence of hox gene expression in the noninternalized ventrolateral NOM in the gastrula, but no axial hox pattern. The first hox gene (Hoxd1) starts expression at the beginning of gastrulation (st. 10.25). Others follow in a temporally collinear sequence during the course of gastrulation and neurulation. Each hox gene is initially expressed in the NOM. There is no early hox expression in the organizer mesoderm (SO). Subsequently, portions of this hox-expressing mesoderm are internalized by a process called involution, and expression of each Hox gene is then initiated in the neurectoderm as well. This is due to a combination of signals from the SO and from the NOM; this combination exclusively being available at the dorsal side of the embryo[19,23]. At the end of gastrulation, there is a spatially collinear axial hox pattern, in the dorsal axial neurectoderm.

## INTERACTIONS BETWEEN THE NONORGANIZER MESODERM AND THE SPEMANN ORGANIZER

It is possible to make dorsalized embryos containing only SO and no NOM by an early treatment of the embryo with lithium chloride at the 64-cell stage. This gives an early hyperactivation of the Wnt pathway and leads to circumferential development of SO mesoderm and no NOM at the gastrula stage[19]. Later on, these embryos become a radially symmetric head without axial trunk structures. They have no hox expression, neither mesodermal nor neural, neither early nor late.

On the other hand, it is possible to make ventralized *Xenopus* embryos, containing only NOM, by UV irradiation of the *Xenopus* zygote (thereby blocking cortical rotation and the subsequent formation of the dorsal SO). The temporally collinear hox expression sequence in the NOM proceeds as normal in the absence of organizer mesoderm (SO) in these ventralized embryos. It has totally normal collinear timing, but it is transient[19]. These embryos later have no axial pattern of hox expression and never show any hox expression in ectoderm[23]. They fail to develop an anterior-posterior axis and become the well-known Bauchstück described in the literature[19,24].

An interaction between the two types of mesoderm is obviously necessary to create a stable anterior-posterior pattern. The SO mesoderm was grafted into ventralized embryos of different ages. This rescued the anterior-posterior axis of the ventralized embryo to different extents according to the age of the NOM. At t = 0 h (both organizer and ventralized embryo at early gastrula stage, st.10), this generated an embryo with an entire embryonic axis and a complete axial pattern of hox expression. If the ventralized embryo was aged for progressively longer times (t = 2, 4, 6 h), before the early SO was implanted, this generated a progressively longer deletion in the axial pattern, starting at the anterior end of the axial hox domain (mid hindbrain level). An experiment using timed blastocelic injection of noggin protein (thereby restoring SO functions) gave a consistent result. Aging the organizer (t = 0, 2, 4, 6 h) transplanted in early UV hosts (t = 0 h) had no effect on the extent of the axial pattern. This indicated that the NOM contains timed information for the axial hox pattern and that the SO does not contain any trunk-patterning information.

## NEURECTODERMAL HOX PATTERN AND VERTICAL SIGNALING

Mangold[24] found evidence for vertical transfer of positional information from the mesoderm to the neurectoderm. It was found that vertical signals from the mesoderm are necessary to induce differentiation of different anterior-posterior levels in the neurectoderm[24]. These signals correspond to Nieuwkoop’s transformation signals[25]. It is likely that the NOM’s hox expression is copied onto the neurectoderm and that Hox gene expression in the NOM regulates vertical signaling to the neurectoderm. The contribution of the NOM is under investigation. Blocking retinoid signaling prevents the mesodermal-neural transfer of Hoxd1[26] and it is likely that this morphogen is required for transfer of anterior hox genes[26,27]. The SO does not deliver detailed positional information for the trunk; however, it is indispensable for formation of a stable anterior-posterior hox pattern. A series of experiments to separate different SO functions was performed to analyze its role in anterior-posterior patterning of the trunk. A knockdown of all organizer functions and restoration of a single one, i.e., neural induction, was sufficient to restore trunk pattern formation. Neural induction (corresponding to Nieuwkoops activation signal[25]) made the ectoderm competent to respond to the patterning signals from the NOM[23].

## THE TIME-SPACE TRANSLATION MODEL

These findings led to a Hox timing model: The time-space translation model (Fig. 1)[19,23,34]. A temporal pattern, appearing in the NOM and indicated by appearance of the collinear sequence of Hox expression, is translated into the ly collinear Hox sequence along the anterior-posterior axis of the neurula stage embryo. This appears in four steps:

1. Hox genes are expressed in a temporally collinear sequence in the NOM. This sequence is transient, as UV ventralized embryos demonstrate.
2. The SO induces (unpatterned) neurectoderm on the dorsal side to make ectodermal cells competent to receive patterning signals.
3. Morphogenetic movements bring the Hox-expressing mesoderm close to the SO in a position beneath the prospective neurectoderm (mesoderm internalization, convergence, and extension). The temporally collinear Hox sequence and the timing of morphogenetic movements enable signaling that leads to the familiar anterior-posterior pattern of Hox gene expression (below).
4. Vertical signals transfer the transient mesodermal Hox expression to the neurectoderm. This generates a stable spatially collinear anterior-posterior Hox pattern in the neurectoderm (and there is possibly also feedback to the mesoderm).

## RELATION TO OTHER MODELS FOR PATTERNING

The simplest interpretation of the results above is that individual hox codes are first sequentially activated in the mesoderm and then transferred from mesoderm to neurectoderm in a time-space translation by copying

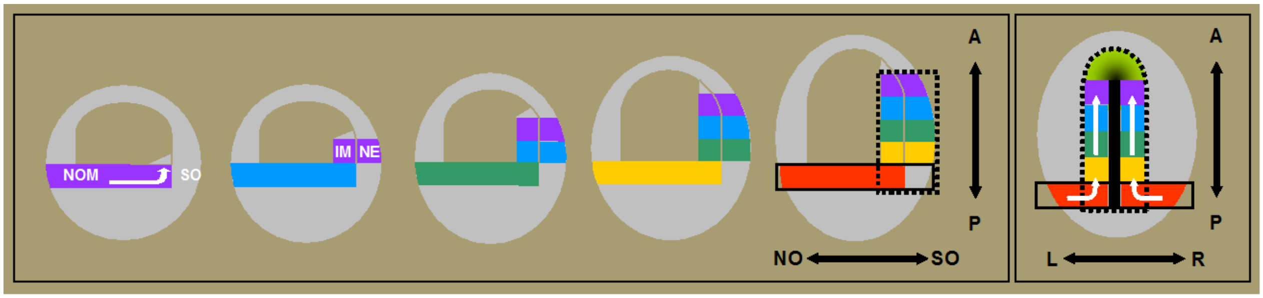

**FIGURE 1.** From Wacker and colleagues[19,23,34]. The drawing shows simplified two-dimensional representations of *Xenopus* gastrulae. The first five drawings show parasagittal (ventral to dorsal) two-dimensional representations of gastrula profiles, starting at the beginning of gastrulation and then at sequential stages until the end. The last (6$^{th}$) drawing shows the end of gastrulation, from the dorsal side (profile at the level of the dorsal axial mesoderm). Hox-expressing tissue (NOM [NO and I] and, late in gastrulation neurectoderm [N]) is represented by different colors, each of which represents a different hox code. Initially, the colored bar represents the broken ring of NOM in the wall of the embryo. The later internal colored blocks at the dorsal side of the embryo represent the involuted NOM. The colored blocks next to them in the wall of the embryo represent the overlying neurectoderm, which also comes to express hox genes. The SO is shown only in the last drawing, as the heavy median black line. By this stage, it has become the notochord and a head mesodermal portion. The first five drawings represent paraxial profiles, where the organizer is not available. The black dotted line depicts the sphere of influence of the SO. Labeling: M: mesoderm; NE: neurectoderm; NOM, NO: nonorganizer mesoderm; SO: Spemann organizer; A: anterior; P: posterior; L: left; R: right. The white arrows reflect directions of cell movement flow. To dorsal, anterior, and internal (drawings 1 and 6). There is a collinear time sequence of hox expression in noninvoluted NOM in the gastrula (depicted by the spectral sequence of colors). During gastrulation, involution movements continuously bring cells from the NOM into the inside of the embryo (see stack of blocks of different colors, reflecting a history of the collinear hox mesodermal time sequence, in the internal involuted mesoderm). Stable (ectodermal) Hox expression is induced by a combination of signals from the SO and the Hox-expressing NOM (see corresponding blocks of sequential spectral colors in the J gastrula's outer layer, resulting from a vertical transfer of the Hox codes from involuted mesoderm to overlying neurectoderm). A "Hox stripe" as part of the anterior-posterior Hox pattern is thus formed at the dorsal side.

hox codes. This should be set against results from others, indicating, for example, that morphogen gradients are important in patterning the axis, e.g., a retinoid gradient patterns the hindbrain[8,27] or a Wnt or FGF gradient patterns the posterior CNS[9]. There is also evidence that a retinoid gradient patterns the presomitic mesoderm[11], and that FGF and RA gradients are general regulators of differentiation in neural tissue and mesoderm[28]. Some of these results are very persuasive. Three comments can be made.

1. The gradient results are generally from later in development (e.g., [4,5]). The axis may be patterned and repatterned at different stages. In fact, we suspect strongly that the mesoderm is repatterned after gastrulation (see above).
2. It is not necessary that every hox code be transmitted from mesoderm to neurectoderm. Maybe there are a few "landmarks" that are established at gastrulation and the details are filled in later. These could, for example, establish the positions of "sources" and "sinks" for axial gradients.
3. Maybe the time-space translation mechanism is complemented by others, possibly working independently of hox genes.

We note that Tschopp et al.[29] showed recently that there are two phases of anterior-posterior patterning and that Hox collinearity is not involved in the second phase. However, their first phase of patterning corresponds to gastrulation.

## CONCLUSIONS

We review experimental investigations and a model indicating that early vertebrate axial patterning is based on Hox temporal collinearity and a time-space translation. This is the first detailed model that uses hox temporal collinearity to generate an axial pattern. We will compare this detailed analysis of the use of hox temporal collinearity as a patterning mechanism to two other existing models (below). Besides these models, there are at least two other timing models in early development. The first is the famous "clock and wavefront" model by Cooke and Zeeman[30], related to vertebrate segmentation and somitogenesis and given a molecular basis by Palmeirim et al.[31]. The second is by Olivera-Martinez and Storey[32]. It acts later in development and deals with pattern temporal regulation of differentiation by Wnt, regulating FGF and retinoid signals. We do not discuss either of these models here because they do not concern the problem of hox temporal collinearity, or deal with hox timing or the regional expression of hox codes.

- **Duboule's "Einbahnstrasse" model** — This model proposes that temporal collinearity coupled with growth control could make an axial pattern[6]. This pioneering proposal, by the discoverer of vertebrate temporal collinearity, is an attractive idea. It could well be relevant in some cases. It has unfortunately never been investigated in detail. It is unlikely that this has relevance to genesis of the *Xenopus* anterior-posterior axial pattern because the tissue that has the temporal collinearity – the gastrula mesoderm – has a low mitotic rate or is nonmitotic[33].
- **Iimura and Pourquié's hox-regulated cell migration model** — Iimura and Pourquié[7] made the exciting discovery that hox expression determines the timing of ingression of chicken gastrula mesoderm cells. Expression of only a 3' hox gene gives early ingression. Add ectopic expression of a 5' hox gene and ingression is delayed considerably. These authors propose that this property, combined with temporal collinearity, makes the chicken's axial hox pattern. Their findings are extremely interesting, but this mechanism cannot generate a pattern without further modification of the cell properties. If temporal collinearity continues during migration in all cells, each cell's hox code will progress to the most posterior value. There can then be no pattern. What is required is stabilization. We propose that hox codes are transferred from involuted/ingressed mesoderm (unstable) to neurectoderm (stable) and that this transfer is regulated precisely. Their findings would then complement our own very nicely. Iimura and Pourquié's findings may also indicate that if a cell's hox code progresses, its migration will stop.
- **The time-space translation model[19,23,34]** — During gastrulation of *Xenopus*, a concerted interaction of temporal gene expression control (i.e., a temporally collinear Hox sequence), morphogenetic movements (i.e., internalization of mesoderm, and convergence and extension), and inductive events (neural induction by SO, vertical transfer from NOM to neurectoderm) functions to create an anterior-posterior pattern in the trunk. This time-space translation mechanism corresponds to the known time course of anterior-posterior patterning in amphibians, predicting anterior-posterior patterning events before neurulation and somitogenesis, and to the fate maps, describing that cells that are internalized early are later located in more anterior regions than cells that are internalized later. This mechanism, which is extremely well supported by the data, represents a novel type of principle for embryonic patterning. Instead of creating a pattern by a morphogen gradient that can act in a static system, time-space translation depends on dynamics that, in this case, are created by the morphogenetic movements of gastrulation and temporal collinearity of the Hox clusters. One can imagine that such a mechanism represents a general developmental principle that is crucial for those organisms that have simultaneous patterning and morphogenesis.
